# Combination of osteopontin and activated leukocyte cell adhesion molecule as potent prognostic discriminators in HER2- and ER-negative breast cancer

**DOI:** 10.1038/sj.bjc.6605840

**Published:** 2010-08-24

**Authors:** M Ihnen, R M Wirtz, K T Kalogeras, K Milde-Langosch, M Schmidt, I Witzel, A G Eleftheraki, C Papadimitriou, F Jänicke, E Briassoulis, D Pectasides, A Rody, G Fountzilas, V Müller

**Affiliations:** 1 Department of Gynecology, University Medical Center Hamburg-Eppendorf, Martinistr. 52, Hamburg D-20246, Germany; 2 Siemens Healthcare Diagnostic Products GmbH, Nattermann Allee 1, Cologne D-50829, Germany; 3 Department of Medical Oncology, Papageorgiou Hospital, Aristotle University of Thessaloniki School of Medicine, Ring Road, Thessaloniki 56403, Greece; 4 Hellenic Cooperative Oncology Group, Data Office, 18 Hatzikonstanti Street, Athens 11524, Greece; 5 Department of Obstetrics and Gynecology, Johannes Gutenberg University, Langenbeckstrasse 1, Mainz D-55101, Germany; 6 Department of Clinical Therapeutics, Alexandra Hospital, University of Athens School of Medicine, 80 Vassilisis Sofias Avenue, Athens 11528, Greece; 7 Department of Medical Oncology, Ioannina University Hospital, Ioannina 45110, Greece; 8 Second Department of Internal Medicine, ‘Attikon’ University Hospital, 1 Rimini Street, Athens 12462, Greece; 9 Department of Obstetrics and Gynecology, JW Goethe University, Theodor-Stern-Kai 7, Frankfurt 60590, Germany

**Keywords:** osteopontin, ALCAM, HER2 and ER-negative breast cancer, discriminative markers

## Abstract

**Background:**

To analyse the discriminative impact of osteopontin (OPN) and activated leukocyte cell adhesion molecule (ALCAM), combined with human epidermal growth factor 2 (HER2) and oestrogen receptor (ER) in breast cancer.

**Methods:**

Osteopontin, ALCAM, HER2 and ER mRNA expression in breast cancer tissues of 481 patients were analysed (mRNA microarray analysis, kinetic RT–PCR). Hierarchical clustering was performed in training cohort A (*N*=100, adjuvant treatment) and validation cohorts B (*N*=200, no adjuvant treatment, low-risk) and C (*N*=181, adjuvant treatment, high-risk).

**Results:**

Negative/low ER and HER2, high OPN and low ALCAM mRNA expression helped to identify patients at particularly high risk, showing shorter DFS, *P*<0.001, and OAS, *P*=0.001. Although both validation cohorts showed diverse risk and treatment profiles, this marker constellation was concordantly associated with shorter DFS and OAS (*P*<0.001 and *P*=0.075 for cohort B and *P*=0.043 and *P*<0.001 for cohort C, respectively). In multivariate analysis, this algorithm was the main independent prognostic factor. Cohort B: DFS, *P*=0.0065, OAS, not significant; cohort C: DFS, *P*=0.026, OAS, *P*<0.001.

**Conclusion:**

Activated leukocyte cell adhesion molecule and OPN mRNA expression has a strong discriminative impact on survival within cancer patients with low or negative expression of ER and HER2, so called ‘high-risk’ breast cancers, and might help in identifying patients who could benefit from new treatment approaches like targeted therapies in the adjuvant setting.

Human breast cancer represents a heterogeneous group of tumours that are diverse in behaviour, outcome and response to therapy. Tumours with hormone receptor and human epidermal growth factor 2 (HER2) negativity are defined as ‘high-risk’ tumours, because of their aggressive growth and resistance to common treatment strategies. However, not all of these tumours behave poorly (Rakha *et al*, 2007). This indicates the underlying heterogeneous nature of these breast cancers, which should therefore be treated by individualised therapy regimens and new treatment approaches in case of assumed therapy resistance. Up to date, all currently available gene signatures have failed to discriminate oestrogen receptor (ESR1)-negative/HER2-negative breast cancer with poor prognosis from those with relatively good outcome (Desmedt *et al*, 2008).

We conducted this study to evaluate the discriminative ability of two experimental markers, osteopontin (SPP1) and activated leukocyte cell adhesion molecule (ALCAM), both regulated by transcription factor Fra-2 (Andersen *et al*, 2002; Milde-Langosch *et al*, 2008), in combination with HER2 and ER. In breast cancer cell lines, OPN has been shown to enhance replication, angiogenesis, evasion from apoptosis, and invasive potential, probably by affecting the expression of several genes, with special reference to the *vascular endothelial growth factor* gene (Cook *et al*, 2005; Chakraborty *et al*, 2008). In breast cancer patients, high OPN protein levels in tumour tissue and blood samples were associated with poor prognosis and disease progression. Beyond that, recent data suggest that patients with OPN overexpression develop predominantly triple-negative tumours (McAllister *et al*, 2008). Despite various published data indicating the negative prognostic effect of OPN in breast cancer, no clinical use has been described yet. Due to its known tumour biological functions, OPN appears to have the ability to identify high-risk tumours, and was therefore included into our model (Rudland *et al*, 2002).

The adhesion molecule ALCAM shows an altered expression in breast cancer, and has been described as a prognostic and predictive marker (Kristiansen *et al*, 2003; King *et al*, 2004; Weichert *et al*, 2004; Swart *et al*, 2005; Verma *et al*, 2005; Burkhardt *et al*, 2006; Ihnen *et al*, 2008). The possibility that ALCAM might be useful in characterising different subsets of breast carcinomas was already indicated by Doane *et al* (2006), who, by genome-wide expression analysis and unsupervised hierarchical clustering, identified two subgroups of ER/PR-negative mammary carcinomas, which differed in the expression of several genes, including *ALCAM*. As *ALCAM* and *SPP1* are characterised by a relatively high dynamic range of expression levels, which were weakly and inversely associated with each other, they were found to be suitable as potential molecular discriminative markers for our study. We performed mRNA microarray analysis and hierarchical cluster analysis, based on *SPP1*, *ALCAM*, *ESR1* and *HER2*, by using cohort A as a training cohort (*N*=100, treated with adjuvant chemotherapy, medium-risk). Based on these results, we generated a decision tree, which was applied to two independent cohorts (cohort B, *N*=200, patients without adjuvant treatment, low-risk, and cohort C, *N*=181, patients treated in the adjuvant setting, high-risk) in order to verify our findings. By this approach, we consistently observed in all the three cohorts that the level of SPP1 and ALCAM mRNA expression enables the discrimination of good *vs* bad outcome in all ‘high-risk’ breast cancer patients showing low or no ESR1 and HER2 expression.

## Materials and methods

### Patients

All patient and tumour characteristics are shown in Table 1. Cohort A showed a normally distributed risk profile. All patients were treated with taxane-free chemotherapy and endocrine therapy according to international recommendations. Cohort B was characterised by a low-risk profile due to node negativity, and therefore the patients did not receive any systemic therapy. Cohort C showed a relatively high-risk profile, characterised by node positivity and/or greater tumour size. The latter patients were treated in the Hellenic Cooperative Oncology Group (HeCOG) 10/97 randomised trial with chemotherapy and endocrine therapy, depending on receptor status. None of the patients within all the three cohorts received trastuzumab. In the first two cohorts, fresh-frozen tissue (FFT) was analysed, whereas in cohort C, formalin-fixed paraffin-embedded (FFPE) material was used.

**Figure 1:**
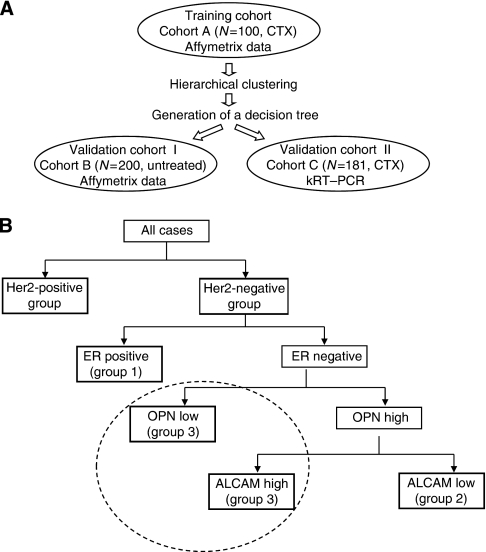
Schematic figure of the experimental design, showing characteristics of all the three cohorts analysed in this study (**A**). Representation of the decision tree, which was generated based on data obtained from the cluster analysis of the training cohort A (**B**).

Informed consent for the scientific use of tissue materials, which was approved by the local ethics committees, was obtained from all patients. The study was performed in accordance to the principles of the declaration of Helsinki and REMARK criteria (McShane *et al*, 2005). Histopathological information was collected from the original pathology reports. The study design is described by Figure 1A.

### Cohort characteristics: cohort A (training cohort)

Fresh-frozen tissue from 100 breast cancer patients treated with adjuvant chemotherapy were collected after surgery, snap-frozen and stored in liquid nitrogen. All patients were treated at the Department of Gynaecology of the University Medical Centre Hamburg Eppendorf, Germany between 1992 and 2002. The median follow-up time was 81 months (range, 7–168 months). No radiotherapy, neoadjuvant chemotherapy or endocrine therapy had been administered before surgery. The patients received the following adjuvant chemotherapy regimens: epirubicin/cyclophosphamide (EC), 37 cases; cyclophosphamide/methotrexate/fluorouracil (CMF), 44 patients; epirubicin (E) or epirubicin/cyclophosphamide/fluorouracil (FEC), 3 cases; and unknown, 16 cases.

### Cohort characteristics: cohort B (validation cohort)

This population-based cohort consisted of 200 consecutive lymph node-negative breast cancer patients, treated at the Department of Obstetrics and Gynaecology of the Johannes Gutenberg University, Mainz between 1988 and 1998. The median age of the patients at surgery was 60 years (range, 34–89 years). The median time of follow-up was 92 months. Patients did not receive any systemic therapy in the adjuvant setting. Patients were treated either with modified radical mastectomy (*N*=75) or with breast-conserving surgery followed by irradiation (*N*=125), and did not show evidence of regional lymph node or distant metastases at the time of surgery.

### Cohort characteristics: cohort C (validation cohort)

Formalin-fixed paraffin-embedded tissues of 181 primary breast cancer patients who were part of the HeCOG 10/97 trial population were collected. The median follow-up time was 97 months. The HeCOG 10/97 trial randomised a total of 595 high-risk (T1-3N1M0 or T3N0M0) breast cancer patients in the period 1997–2000, to receive either four cycles of E followed by four cycles of intensified CMF (E-CMF) or three cycles of epirubicin followed by three cycles of paclitaxel (T) and three cycles of intensified CMF (E-T-CMF), as previously described (Fountzilas *et al*, 2005).

### RNA isolation from FFT (cohorts A and B)

Approximately, 50 mg of fresh-frozen breast tumour tissue was crushed in liquid nitrogen. Tumour cell content exceeded 40% in all the samples, as shown by H&E staining of cryo-cut sections. RLT-Buffer (QIAGEN, Hilden, Germany) was added and the homogenate was centrifuged through a QIAshredder column (QIAGEN). From the eluate, total RNA was isolated using the RNeasy Kit (QIAGEN) according to the manufacturer's instructions. The RNA yield was determined by UV absorbance and its quality was assessed by evaluating the ribosomal RNA band integrity on an Agilent 2100 Bioanalyzer RNA 6000 LabChip kit (Agilent Technologies, Palo Alto, CA, USA).

### Microarray analysis (cohorts A and B)

The Affymetrix (Santa Clara, CA, USA) HG-U133A array and GeneChip System were used to quantify the relative transcript abundance in the breast cancer tissues. Starting with 5 *μ*g total RNA labelled cDNA was prepared using the Roche Microarray cDNA Synthesis (Roche Diagnostics GmbH, Roche Applied Science, Mannheim, Germany), Microarray RNA Target Synthesis (T7) and Microarray Target Purification Kit, according to the manufacturer's instructions. In the analysis settings, the global scaling procedure was chosen, which multiplied the output signal intensities of each array to a mean target intensity of 500. Samples with suboptimal average signal intensities (i.e., scaling factors >25 or GAPDH 3′/5′ ratios >5) were re-labelled and re-hybridised on new arrays.

### RNA isolation from FFPE tissue and kRT–PCR assessment (cohort C)

Intact RNA with high quality, as determined by analysis of the housekeeping gene *RPL37A*, was isolated from 181 FFPE samples from the HeCOG cohort by using an experimental method based on proprietary magnetic beads from Siemens Healthcare Diagnostics Products GmbH (Cologne, Germany), as previously described (Pentheroudakis *et al*, 2009). In total, 98 patients were treated with E-CMF and 83 with E-T-CMF. The number of malignant cells represented at least 30% of all nucleated cells per section, as verified by H&E staining. Kinetic reverse transcription-polymerase chain reaction (kRT–PCR) was applied for the assessment of mRNA expression of *ALCAM*, *SPP1*, *HER2* and *ESR1*, using the following TaqMan-based primer/probe sets:

ALCAM probe 5′-CCTTGCCGCAAAGTGTGTAACGGAAT-3′

Forward primer 5′-CGCAAGTGTAAGAAGTGCGAA-3′

Reverse primer 5′-CGTAGCATTTATGGAGAGTGAGTCT-3′

SPP1 probe 5′-CTCAAAGGTACTCCCTCCTCCCGGG-3′

Forward primer 5′-CGGTTATGTCATGCCAGATACAC-3′

Reverse primer 5′-GAACTGAGACCCACTGAAGAAAGG-3′

HER2 probe 5′-ACCAGGACCCACCAGAGCGGG-3′

Forward primer 5′-CCAGCCTTCGACAACCTCTATT-3′

Reverse primer 5′-TGCCGTAGGTGTCCCTTTG-3′

ESR1 probe 5′-CACAGACTGCTTTGCCTGCATGAATTTC-3′

Forward primer 5′-GAGGCTGCTCAGGACCTAAGG-3′

Reverse primer 5′-GAGTAACACATGCTCCACTGTCATT-3′

Forty cycles of amplification were applied and the cycle threshold (CT) values of the target genes were identified. Cycle threshold values were normalised by subtracting the CT value of the housekeeping gene *RPL37A* from the CT value of the target gene (ΔCT). RNA results were then reported as 40−ΔCT values, which would correlate proportionally to the mRNA expression level of the target gene.

Human reference total RNA pooled from 10 human cell lines (Stratagene, La Jolla, CA, USA) was used as a positive control. RNA-free DNA extracted from tumour tissues was used as a negative control.

### Statistical analysis

Fisher's exact test was applied to compare the clinical and pathological factors with molecular gene expression (low *vs* high). Spearman's rank correlation was used as a measure of association between variables. Time to event distributions were estimated using Kaplan–Meier curves and compared using the log-rank test.

Disease-free survival was defined as the interval from study entry to disease recurrence or death from any cause. Overall survival was measured from study entry until death from any cause. Surviving patients were censored at the date of last contact. For prognosis evaluation the following variables were included into the analysis of cohort A: age (<52 years *vs* 52 years and older), tumour size (<2 cm *vs* 2–5 cm *vs* >5 cm), tumour grade (I–II *vs* III), histological type (ductal *vs* lobular *vs* others), nodal involvement *vs* nodal-negative tumours, and immunohistochemical ER and PR status (negative *vs* positive). In cohort B, age at diagnosis (<60 years *vs* the median age of 60 years and older), tumour size (⩽2 cm *vs* >2 cm), immunohistochemical ER, PR and HER2 status (negative *vs* positive), and tumour grade (I *vs* II *vs* III) were compared. In the analysis of cohort C, we included the randomisation group (E-T-CMF *vs* E-CMF), age, tumour size (<2 cm *vs* 2–5 cm *vs* >5 cm), histological type (ductal *vs* lobular *vs* mixed *vs* other), adjuvant endocrine treatment (yes *vs* no), radiotherapy (yes *vs* no), ER/PR status (negative *vs* positive), as well as menopausal status (pre *vs* post), number of positive nodes (0–3 *vs* ⩾4) and tumour grade (I–II *vs* III-undifferentiated) into our correlations. The Cox proportional hazards model was used to assess the strength of the association of OAS and DFS with clinical and histological variables in the presence of group classification. Backward selection procedure was used, with removal criterion *P*>0.10, to identify a subclass of significant clinical variables. The level of statistical significance was *P*=0.05 for all tests. All *P*-values were two-sided. The results of this study were presented according to the REMARK criteria for tumour marker studies (McShane *et al*, 2005). The statistical analysis was conducted using SPSS 15.0 for windows or JMP 5.0.1.2 programs. Hierarchical cluster analysis, decision tree model and Kaplan–Meier analysis in cohorts A and B were performed using the JMP 5.0.1.2 program. For the initial cluster analysis in the finding cohort, the *ESR1* and *HER2* expression values were scaled down by a factor of 5 compared with the *ALCAM* and *SPP1* values to reduce their corruptive effect on data analysis because of their relatively high expression levels. For separation of all HER2-positive cases from clusters I–III in cohort A, we used a cutoff value of 6000 at TGT500. For the decision tree model in cohort B, we used predefined cutoffs of 6000 for *HER2* and 1200 for *ESR1*. For the distinction of *SPP1* and *ALCAM* levels in cohort B, we used the objective 50th percentile in order to define low and high mRNA expression of *ALCAM* and *SPP1* (predefined cutoffs for *SPP1* was 2181.0 and for *ALCAM* was 3193.5).

As for cohort C, predefined cutoffs were used for *ESR1* and *HER2* mRNA expression, which were close to the 25th and 75th percentile, respectively. The median normalised ΔCT value for *ESR1* was 35.58 (range, 28.51–40.30), for *HER2* was 35.48 (range, 30.35–41.51), for *ALCAM* was 34.55 (range, 27.09–37.09) and for *SPP1* was 31.38 (range, 26.52–41.95).

## Results

### Cohort A: hierarchical cluster analysis of the training cohort

**Figure 2:**
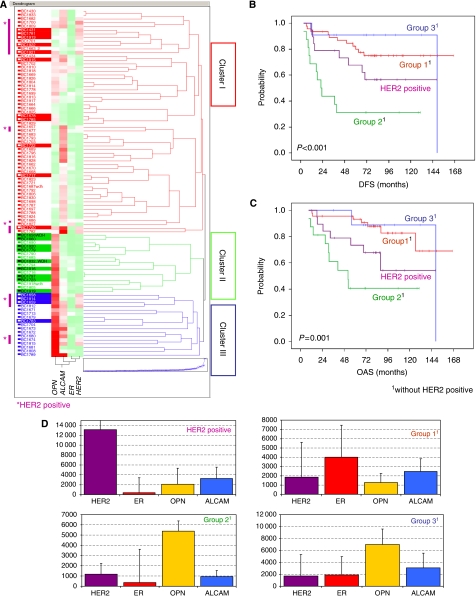
Cohort A (training cohort) hierarchical cluster analysis based on *OPN (SPP1)*, *ALCAM*, *ER (ESR1)* and *HER2* mRNA expression levels (**A**), revealing three main clusters (I–III). In the coloured map, gene expression levels ranged from low (green), to moderate (white), to high (red). Cases with recurrences during follow-up are highlighted with coloured horizontal bars on the left. Tumours with high *HER2* expression are marked on the left margin of the graph (purple bars). Based on clusters I–III and after separating HER2-positive cases, four groups (groups 1–3 and the HER2-positive group) were identified (**D**) (median values and s.d are given). (**B**) and (**C**) show Kaplan–Meier curves for DFS and OAS of the four groups, with group 2 being the ‘high-risk’ group characterised by ER/HER negativity, high OPN and low ALCAM mRNA expression.

On the basis of Ward correlation, a hierarchical cluster analysis was performed. Focused on *SPP1*, *ALCAM*, *HER2* and *ESR1* mRNA expression, the analysis revealed three main clusters (Figure 2A). Cluster I was characterised by relatively high *ESR1* expression, variable *HER2* and *ALCAM* expression, and weak or negative *SPP1* expression.

Cluster II was characterised by predominantly negative *ESR1* and *HER2* expression, high *SPP1*, and weak or negative *ALCAM* expression.

Cluster III showed variable *ESR1* and *HER2* expression and positive *SPP1* and *ALCAM* expression levels. When we analysed these clusters with respect to the occurred events, patients who suffered from recurrence were predominantly found in cluster II.

In clusters I and III the recurrence rates were lower, and most of the events occurred in cases expressing high *HER2* mRNA levels (Figure 2A, purple bars on the left side).

We therefore separated the HER2-positive cases from clusters I–III to build a HER2-positive group, containing all HER2-positive cases, and we designated three other groups (groups 1–3), which were based on our clusters henceforth containing only cases with low/negative *HER2* expression. Each of these groups showed a specific distribution of ER, HER2, ALCAM and OPN expression (Figure 2D).

When we performed a Kaplan–Meier analysis including all the four groups (groups 1–3 and the HER2-positive group), group 2 (characterised by weak or negative ER and HER2 expression, and high OPN and low ALCAM expression) turned out to contain predominantly high-risk cases with highly significant differences in DFS (*P*<0.001) and OAS (*P*=0.001) compared with all the other groups (Figure 2B and C, respectively). A detailed pairwise analysis within all the groups revealed further significant differences for DFS and OAS. These results are shown in Table 2a.

### Cohort B: mRNA microarray analysis and decision tree application

**Figure 3:**
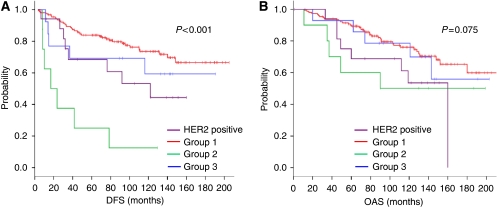
Kaplan–Meier curves of cohort B (first validation cohort) showing DFS (**A**) and OAS (**B**) in groups 1–3 and the HER2-positive group classified by their *HER2*, *ER*, *OPN* and *ALCAM* expression levels as follows: HER2 pos.: HER2-positive tumours; group 1: HER2-negative/ER-positive tumours; group 2: ER/HER2-negative, OPN-high, ALCAM low tumours (high-risk group); group 3: ER/HER2-negative, OPN-low tumours or OPN-high/ALCAM-high tumours.

Based on the results of the hierarchical clustering in cohort A, we generated a decision tree model (Figure 1B). By this approach, we defined four different groups within cohort B: the HER2-positive group: *n*=20 (HER2-positive tumours); group 1: *n*=156 (HER2-negative, ER-positive tumours with intermediate ALCAM and low OPN expression); group 2: *n*=14 (HER2/ER-negative tumours with high OPN and low ALCAM expression); group 3: *n*=10 (HER2/ER-negative tumours with predominantly higher OPN and ALCAM expression). Kaplan–Meier curves for DFS (Figure 3A) differed significantly (*P*<0.001), while the differences in OAS showed a trend of being significant between all groups (*P*=0.075; Figure 3B). Statistically significant differences in pairwise analysis between all generated groups are presented in Table 2b. By multivariate analysis including grading, tumour size and ER immunohistochemical (IHC) status, the decision tree classification was shown to be the only significant independent predictor of DFS (*P*=0.0065), whereas histological grading (*P*=0.060), ER status (*P*=0.107) and tumour size (*P*=0.235) lost their significance (data not shown). When comparing the *SPP1* mRNA expression levels with clinicopathological data, no significant associations with immunohistochemically determined ER, PR or HER2 status, clinical stage, age, and histological grading were found (data not shown). In contrast, significant positive correlations of high *ALCAM* expression levels with low grading (*P*=0.009), smaller tumour size (*P*=0.023) and positive PR (IHC) results (*P*=0.027) were obtained, whereas the association with ER (IHC) positivity did not reach statistical significance (*P*=0.101).

### Cohort C: kRT–PCR and decision tree application

**Figure 4:**
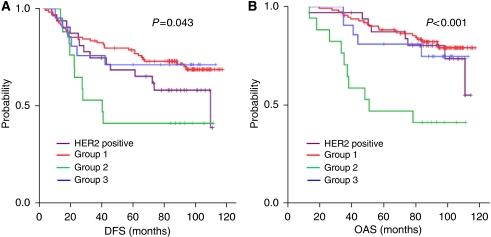
Kaplan–Meier curves of cohort C (second validation cohort) showing DFS (**A**) and OAS (**B**) in groups 1–3 and the HER2-positive group classified by their HER2, ER, OPN and ALCAM expression levels as follows: HER2 pos., HER2-positive tumours; group 1: HER2-negative/ER-positive tumours; group 2: ER/HER2-negative, OPN-high, ALCAM-low tumours (high-risk group); group 3: ER/HER2-negative, OPN-low tumours or OPN-high/ALCAM-high tumours.

In order to test the discriminative value of our algorithm in FFPE tissue, the four markers were analysed in cohort C by using kRT–PCR. As former studies have shown that mRNA quantification using mRNA microarray analysis and RT–PCR resulted in similar gene expression levels (Modlich *et al*, 2004; Zamagni *et al*, 2009), we found it reasonable to use cohort C for verification. Based on the same decision tree algorithm that we used in cohort B (Figure 1B), 32 tumours were classified in the HER2-positive group, 111 in group 1 (HER2-negative, ER-positive tumours with intermediate ALCAM and low OPN), 17 in group 2 (HER2/ER-negative tumours with high OPN and low ALCAM expression) and 21 in group 3 (HER2/ER-negative tumours with predominantly higher OPN and ALCAM expression). Kaplan–Meier curves for DFS (Figure 4A) and OAS (Figure 4B) differed significantly between all the four groups (DFS, *P*=0.043 and OAS, *P*<0.001, respectively). For pairwise comparisons see Table 2c.

When groups 1, 3 and the HER2-positive group were combined and compared with group 2, significant differences for DFS (*P*=0.013) and OAS (*P*=0.001) were observed (data not shown).

The discriminative value of our gene-set algorithm was also examined with regard to patients, who were randomised to the paclitaxel- or non-paclitaxel-containing treatment arm in the HeCOG 10/97 trial. Patients whose tumours showed ER/HER2 negativity, and high OPN and low ALCAM expression (group 2) turned out to be the group having the highest risk for shorter DFS and OAS compared with all the other groups regardless of the administered therapy (see Table 2d and e). Multivariate analysis revealed that only the algorithm classification and the number of positive nodes (⩾4) are independent predictors of outcome in this cohort (Table 3). Group 2 was associated with an increased risk of death (HR=3.94, 95% CI 1.92–8.09, *P*<0.001) and relapse (HR=2.18, 95% CI 1.10–4.34, *P*=0.026). Four or more positive nodes were associated with shorter OAS (HR=3.21, 95% CI 1.14–9.01, *P*=0.027) and DFS (HR=2.21, 95% CI 1.05–4.65, *P*=0.038).

Comparison of *ALCAM* expression with clinicopathological data did not show any significant associations (data not shown). *Osteopontin* expression was not found to be associated with ER or HER2, nor with any other clinicopathological factors.

## Discussion

The results of the present hypothesis-generating study show that the application of the two, in this context newly discovered, molecular markers, ALCAM and OPN, could discriminate prognostic subgroups within HER2- and ER-negative early breast cancer patients. These results were detectable regardless of the administered adjuvant therapy regimens or patients’ risk profiles, and might therefore help identify receptor-negative breast cancer patients characterised by a particular unfavourable outcome. Although we used different cohorts and analysed gene expression by two methods, we were able to show this effect in each of these three cohorts, which to our opinion strengthens our algorithm, even though our results have to be validated in a larger cohort with a prospective design. We became interested in ALCAM and OPN in the course of our previous studies on the AP-1 protein Fra-2, as both genes are regulated by this transcription factor (Andersen *et al*, 2002; Milde-Langosch *et al*, 2008). Based on previous OPN studies and own findings regarding the role of ALCAM in breast cancer (Ihnen *et al*, 2008), we decided to evaluate the discriminative value of these genes in combination with *HER2* and *ESR1* in an effort to identify breast cancer subgroups, in which *SPP1* and *ALCAM* are of particular relevance as we attributed to these genes a certain discriminative impact.

The *HER2* gene amplification and/or protein overexpression is found in 15–30% of all invasive breast cancers and has been associated with more aggressive disease and shorter disease-free survival, through activation of several intracellular pathways ultimately affecting cell proliferation, survival, motility and adhesion (Baselga *et al*, 2005). Based on our hierarchical cluster analysis we excluded all HER2-positive cases from each cohort to analyse the outcome in this group separately, taking into account the high impact of HER2 overexpression on survival. As expected, an unfavourable prognosis was seen in the HER2-positive group, but patients expressing no HER2 but high OPN and low ALCAM (group 2) were found to have an even worse survival (see Figures 2, 3 and 4).

Regarding the ER expression status, it is well known that high ER expression is associated with beneficial prognosis. In our study, improved survival was seen in patients whose tumours expressed high ER levels, as shown in cluster I and concordantly in group 1, within all the three cohorts (see Figures 2, 3 and 4). To date, it is standard to determine the ER expression status by IHC methods. Recent findings have shown that RT–PCR determination of ER expression is superior to ligand binding or IHC approaches for prediction of distant recurrence-free survival (Kim *et al*, 2006). Referring to that, we saw that ER determination by kRT–PCR correlates moderately with IHC results (Kim *et al*, 2006; Pentheroudakis *et al*, 2009). Based on different gene expression levels, we obtained besides the HER2 positive. group and group 1, another two additional groups (group 2 and group 3) (see Figures 2, 3 and 4). Group 3 showed low ER and HER2 and high OPN and ALCAM expression levels. Group 2 was characterised by the same expression pattern, but showed, unlike group 3, low ALCAM expression levels. Group 3 exhibited survival rates similar to those observed in the favourable prognosis group 1; group 2 showed the poorest outcome compared with all the groups. These observations appear to suggest that *ALCAM*, or some co-regulated gene, has beneficial effects on receptor-negative and high *SPP1*-expressing tumours. Although high ALCAM expression levels have been associated with better outcome and might be predictive for chemotherapy response in breast cancer, the underlying biological mechanism for survival benefit still remains unclear (King *et al*, 2004; Ihnen *et al*, 2008). As known, OPN expression has been associated with worse outcome and aggressive tumour growth. Our observations, however, indicate that high OPN expression *per se* may not necessarily function as a driving force of increased aggressiveness, as shown in group 3. It might be possible that OPN shows adverse potential in combination with specific marker expression patterns, like low ALCAM expression levels. The previous observation that OPN expression seems to be associated with the increased appearance of predominantly triple-negative tumours (Rudland *et al*, 2002; Cook *et al*, 2005; McAllister *et al*, 2008) appears to be reflected by our results showing that ER- and HER2-negative tumours predominantly express high OPN levels (see Figure 2A, cluster II and Figure 2D). Of note, our experimental study design does not discriminate between splice variants or the origin of the OPN mRNA expression. This means it remains unclear whether OPN is expressed by tumour cells, stromal fibroblasts or inflammatory cells.

As regard the technical aspect of this study, it should be added that the comparability of mRNA microarray data (cohorts A and B) with RT–PCR data (cohort C) regarding gene expression levels might be a subject of discussion. But as other studies have shown that the RNA quantification by using mRNA microarray analysis and RT–PCR resulted in similar gene expression levels (Modlich *et al*, 2004; Zamagni *et al*, 2009), we deemed it appropriate to include the RT–PCR results of cohort C into our study as a second verification cohort. Although we agree that the comparability of mRNA expression with protein expression levels might be of interest and could be a subject of discussion, we refrained from including western blot and IHC methods into this study. Our group and others have shown that there is a good correlation between ER, HER2 and ALCAM mRNA expression levels compared with the existing results of IHC and western blot expression (Kim *et al*, 2006; Ihnen *et al*, 2008; Pentheroudakis *et al*, 2009). This observation might lead to the assumption that mRNA expression levels of these markers could function as prognostic and predictive markers by themselves. Regarding the most appropriate OPN detection method, *SPP1* mRNA expression analysis appears to be advantageous, as evaluation of OPN protein expression is constrained by the large number of OPN splice variants, phosphorylation and glycosylation products, as well as by differences in specificity of the available antibodies towards these protein forms (Kon *et al*, 2000). These reasons led us to the decision to focus on *SPP1*, *ALCAM*, *ESR1* and *HER2* mRNA expression in our study.

In conclusion, our marker set algorithm might be instrumental in identifying receptor-negative patients who are suffering from particularly high-risk tumours that are unlikely to respond to therapy regimens as used in our cohorts. These patients could be candidates for new therapeutic approaches, like targeted therapies or intensive chemotherapy. Our findings need to be validated in a larger prospective study, directly comparing mRNA microarray and kRT–PCR in FFT and FFPE tumour tissues, as kRT–PCR appears to be a valid and feasible method for quantification of gene expression and could provide the advantage of being more applicable in clinical routine use.

## Figures and Tables

**Table 1:** Patient characteristics

	**Cohort A (training) *N*=100**	**Cohort B (validation) *N*=200**	**Cohort C (validation) *N*=181**
	***n* (%)**	***n* (%)**	***n* (%)**
*Age (years)*			
Median	52	60	52
Range	29–73	34–89	22–76
			
*Menopausal status*			
Pre-/peri-menopausal	63 (63)	—	90 (50)
Post-menopausal	24 (24)	—	91 (50)
Unknown	13 (13)	—	0
			
*Histological type*			
Ductal	73 (73)	127 (64)	136 (75)
Lobular	15 (15)	35 (18)	22 (12)
Others	11 (11)	15 (13)	23 (13)
Unknown	1 (1)	23 (12)	0
			
*Tumour size (stage)*			
⩽2 cm (pT1)	24 (24)	112 (56)	57 (32)
2–5 cm (pT2)	63 (63)	85 (43)	89 (49)
>5 cm (pT3–4)	12 (12)	3 (2)	35 (19)
Unknown	1 (1)	0	0
			
*Grade*			
I–II	39 (39)	163 (82)	86 (48)
III–undifferentiated	59 (59)	37 (19)	94 (52)
Unknown	2 (2)		1 (1)
			
*Lymph nodes*			
Positive nodes	42 (42)	0	178 (98)
1–3	—	0	39 (22)
⩾4	—	0	139 (77)
Negative nodes	58 (58)	200 (100)	3 (2)
			
*ER status*			
Positive	65 (65)	163 (82)	128 (71)
Negative	32 (32)	37 (19)	50 (28)
Unknown	3 (3)	0	3 (2)
			
*PR status*			
Positive	54 (54)	144 (72)	109 (60)
Negative	43 (43)	56 (28)	66 (36)
Unknown	3 (3)	0	6 (3)
			
*HER2 status*			
Positive	—	26 (13)	53 (29)
Negative	—	165 (83)	124 (68)
Intermediate	—	9 (5)	—
Unknown	100 (100)	0	4 (2)
			
*Adjuvant RT*			
Yes	58 (58)	125 (63)	141 (78)
No	19 (19)	75 (37)	39 (22)
Unknown	23 (23)	0	1 (1)
			
*Adjuvant HT*			
Yes	54 (54)	0	168 (93)
No	35 (35)	200 (100)	11 (6)
Unknown	11 (11)	0	2 (1)
			
*Follow-up*			
Recurrence	33 (33)	58 (29)	55 (30)
Died of disease	20 (20)	57 (29)	37 (20)

Abbreviations: ER=oestrogen receptor; HER2=human epidermal growth factor 2; HT=hormone therapy; PR=progesterone receptor; RT=radio therapy.

**Table 2:** Pairwise comparison of DFS and OAS in groups 1–3 and the HER2-positive group generated by our algorithm

	**HR**	**95% CI**	***P***
*(a) Cohort A*			
DFS			
Group 2 *vs* group 1	4.58	1.96–10.67	<0.001
OAS			
Group 2 *vs* group 1	5.76	2.10–15.77	0.001
Group 2 *vs* group 3	9.01	1.13–71.40	0.038
HER2 pos. *vs* group 1	1.43	1.01–2.03	0.047
			
*(b) Cohort B*			
DFS			
Group 2 *vs* HER2 positive.	1.79	1.07–3.00	0.020
Group 2 *vs* group 1	8.25	3.62–18.79	<0.001
Group 2 *vs* group 3	3.79	1.18–12.20	0.017
			
*(c) Cohort C*			
DFS			
Group 2 *vs* group 1	2.58	1.26–5.25	0.009
OAS			
Group 2 *vs* HER2 positive.	3.55	1.40–9.01	0.008
Group 2 *vs* group 1	4.71	2.21–10.04	<0.001
			
*(d) Cohort C (paclitaxel group)*			
OAS			
Group 2 *vs* group 1	4.44	1.20–16.47	0.026
			
*(e) Cohort C (non-paclitaxel group)*			
DFS			
Group 2 *vs* group 1	3.24	1.32–7.94	0.010
Group 2 *vs* group 3	5.42	1.12–26.25	0.036
OAS			
Group 2 *vs* HER2 positive.	3.30	1.02–10.72	0.047
Group 2 *vs* group 1	5.08	1.97–13.09	0.001
Group 2 *vs* group 3	12.55	1.54–102.67	0.018

Abbreviations: CI=confidence interval; DFS=disease-free survival; HER2=human epidermal growth factor 2; HR=hazards ratio; OAS=overall survival.

Only significant differences are given for all three cohorts (A, B and C).

**Table 3:** Cox regression analysis including conventional prognostic markers and groups 1–3 and the HER2-positive group of cohort C based on our four-gene algorithm

	**HR**	**95% CI**	***P***
*(a) OAS*			
Grade			
I–II	1		
III—undifferentiated	1.72	0.92–3.30	0.086
Positive nodes			
0–3	1		
⩾4	3.21	1.14–9.01	0.027
Algorithm classification			
Group 1+3+HER2 positive.	1		
Group 2	3.94	1.92–8.09	<0.001
Treatment group			
E-T-CMF	1		
E-CMF	1.18	0.64–2.17	0.603
			
*(b) DFS*			
Grade			
I–II	1		
III–undifferentiated	1.45	0.87–2.42	0.157
Positive nodes			
0–3	1		
⩾4	2.21	1.05–4.65	0.038
Algorithm classification			
Group 1+3+HER2 positive.	1		
Group 2	2.18	1.10–4.34	0.026
Treatment group			
E-T-CMF	1		
E-CMF	1.08	0.65–1.80	0.767

Abbreviations: CI=confidence interval; DFS=disease-free survival; E-CMF=epirubicin-cyclophosphamide, methotrexate and fluorouracil; E-T-CMF=epirubicin-paclitaxel-cyclophosphamide, methotrexate and fluorouracil; HER2=human epidermal growth factor 2; HR=hazards ratio; OAS=overall survival.

Only the presented algorithm and the number of positive nodes (⩾4) are independent predictors of outcome in this cohort.
